# Physical and Enzymatic Hydrolysis Modifications of Potato Starch Granules

**DOI:** 10.3390/polym14102027

**Published:** 2022-05-16

**Authors:** Nasima Chorfa, Hervé Nlandu, Khaled Belkacemi, Safia Hamoudi

**Affiliations:** Department of Soil Sciences and Agri-Food Engineering, Centre in Green Chemistry and Catalysis, Laval University, Quebec City, QC G1V 0A6, Canada; nasima.chorfa@fsaa.ulaval.ca (N.C.); herve.nlandu.1@ulaval.ca (H.N.); khaled.belkacemi@fsaa.ulaval.ca (K.B.)

**Keywords:** potato starch, supercritical CO_2_, high-energy ball milling, ultrasonication, enzymatic hydrolysis, Pullulanase

## Abstract

In this work, a valorization of the starch stemming from downgraded potatoes was approached through the preparation of starch nanoparticles using different physical methods, namely liquid and supercritical carbon dioxide, high energy ball milling (HEBM), and ultrasonication on the one hand and enzymatic hydrolysis on the other hand. Starch nanoparticles are beneficial as a reinforcement in food packaging technology as they enhance the mechanical and water vapor resistance of polymers. Also, starch nanoparticles are appropriate for medical applications as carriers for the delivery of bioactive or therapeutic agents. The obtained materials were characterized using X-ray diffraction as well as scanning and transmission electron microscopies (SEM and TEM), whereas the hydrolysates were analyzed using size exclusion chromatography coupled with pulsed amperometric detection (SEC-PAD). The acquired results revealed that the physical modification methods led to moderate alterations of the potato starch granules’ size and crystallinity. However, enzymatic hydrolysis conducted using Pullulanase enzyme followed by nanoprecipitation of the hydrolysates allowed us to obtain very tiny starch nanoparticles sized between 20 and 50 nm, much smaller than the native starch granules, which have an average size of 10 μm. The effects of enzyme concentration, temperature, and reaction medium pH on the extent of hydrolysis in terms of the polymer carbohydrates’ fractions were investigated. The most promising results were obtained with a Pullulanase enzyme concentration of 160 npun/g of starch, at a temperature of 60 °C in a pH 4 phosphate buffer solution resulting in the production of hydrolysates containing starch polymers with low molecular weights corresponding mainly to P-10, P-5, and fractions with molecular weights lower than P-5 Pullulan standards.

## 1. Introduction

Agri-food sector activities generate large volumes of various by-products which must be disposed of judiciously to preserve the environment on the one hand and to give added value to this biomass on the other hand. This is the case with downgraded potatoes, rich in starch, which represents a valuable resource for a successful industrial recovery. Starch is a polysaccharide composed of glucose units, stored in several plants’ reserve organs such as cereals (30–70% of dry matter), tubers (60–90%), and legumes (25–50%). It is the major source of carbohydrates in animal and human food [1]. Starch is also used in many non-food industrial sectors such as paper, pharmaceuticals, cosmetics, textiles, etc. [2]. In recent years, it has also become an interesting raw material to produce bio-based and biodegradable materials as well as bioethanol [1].

Starch consists of two homopolymers of *α*-D-glucopyranose: (i) amylose, a linear polymer composed of glucose units linked by *α*-(1–4) bonds, and (ii) amylopectin, a highly branched polymer made of glucose units with *α*-(1–6) bonds [3]. Potato starch is composed of 21% amylose and 79% amylopectin; it occurs naturally in the form of insoluble, semi-crystalline granules. Each granule has a layered organization with alternating amorphous and semi-crystalline growth rings of similar thicknesses of 120–400 nm [4,5]. The branches of amylopectin within the granules are often arranged as double helices (A-type or B-type) and are located in the crystalline lamellae, whereas the amorphous lamellae mostly contain amylose, branch points, and chains not organized as double helices [6]. Starch also contains compounds in minor quantities, such as lipids, proteins, and phosphorus compounds, whose proportions depend on the botanical origin.

After extraction, native potato starch granules are in the form of a white powder that is insoluble in cold water and their grain size varies between 5 and 100 μm [7]. In fact, these granules are insoluble in any conventional solvent at room temperature despite being highly hydrophilic. However, when suspended and heated in excess water, starch undergoes an order–disorder transition called gelatinization [8]. During this phenomenon, starch granules swell, amylose progressively leaches out of the granules, and the semi-crystalline structure is disrupted. Generally, the disruption of the semi-crystalline structure of starch granules leads to the formation of starch nanoparticles [9]. Such particles are useful in several applications. For instance, starch nanoparticles can enhance the mechanical and water vapor resistance of a polymer to be utilized in food packaging technology [10]. Also, starch nanoparticles are suitable for medical applications since they can be used as carriers for the delivery of immobilized bioactive or therapeutic agents [11]. In addition, organically functionalized starch nanoparticles were successfully used for the removal of several pollutants in wastewater treatment applications [12].

Several methods were employed for the preparation of starch nanoparticles with different physicochemical and mechanical properties [9]. Among them, are supercritical CO_2_ treatment, ultrasonication, and high-energy ball milling [13,14]. In addition, such treatments were also used to prepare the starch granules for a subsequent hydrolytic treatment with the objective of producing monodisperse starch nanoparticles [15].

Supercritical CO_2_ is the most used supercritical fluid because it has moderate critical conditions, can readily be separated from solutes, poses no environmental problems, and is non-flammable, non-toxic, as well as inexpensive. The low critical temperature (304.15 K) and pressure (72.8 atm) of CO_2_ make it the ideal solvent for natural products, which tend to be susceptible to thermal degradation during processing [16].

Ball milling entails several mechanical actions encompassing collision, friction, shearing, impingement, and grinding. It is regarded as an environmentally friendly and green non-chemical low-cost treatment [17,18]. Ball milling was previously reported to be able to modify the morphology, crystallinity, and molecular weight of starch granules of different origins [14,19,20,21,22].

High-intensity ultrasound is a physical treatment without any chemical reaction. Recently, it has attracted great interest in many food applications, such as emulsifying, sterilizing, extracting, degassing, filtrating, drying, and enhancing oxidation [23,24]. High-intensity ultrasound generated by periodic mechanical motions of a probe, transfers ultrasonic energy into a fluid medium and triggers extremely high alterations in pressure leading to the formation of small rapidly growing bubbles (cavities) [25], which expand during the negative pressure step and implode violently during the positive step generating high temperatures, pressures, and shear forces at the probe tip [26]. This ecofriendly approach is an alternative solution for reducing the processing time to generate starch nanoparticles and increasing the yield production [27,28]. Ultrasonication is useful for modifying the physicochemical and functional properties of starch and has many advantages in terms of higher selectivity and quality, less chemical usage, and short processing duration as reported by Rahaman et al. [29] as well as Hedayati et al. [30]. For instance, Bel Haaj et al. [28] reported the complete conversion of the starch granules from micron to nanoscale after 75 min of ultrasonication at a temperature of 8–10 °C.

Acid hydrolysis has been used to modify starch granule structure and produce “soluble starch” or glucose syrup for many years [31]. The limiting factors for the wide use of acid hydrolysis are related to the use of toxic acids, the slow reaction, and the random hydrolytic action of the acid which does not allow the control of the size of the resulting starch particles. On the contrary, enzymatic hydrolysis offers the advantage of being faster with higher yields in comparison to acid hydrolysis [9]. Indeed, the use of active enzymes to modify carbohydrates is an extremely selective and versatile biotechnological tool.

The present investigation focuses on the valorization of potato starch from downgraded potatoes through the preparation of starch nanoparticles using different methods, namely supercritical carbon dioxide (ScCO_2_), high energy ball milling (HEBM), ultrasonication (US), as well as enzymatic hydrolysis. The investigation aims to understand how the selected treatment method influences the molecular weight and the structural changes of starch granules.

## 2. Materials and Methods

### 2.1. Materials

The downgraded potatoes used in this investigation belong to the “Gabrielle” variety. They were graciously provided by “La Ferme Valupierre” (Quebec, QC, Canada). Pullulanase enzyme (E2412) and a phosphate buffer solution at a pH of 4 were acquired from Sigma-Aldrich and used without further purification.

### 2.2. Starch Extraction and Treatments

Starch extraction from the downgraded potatoes was conducted as reported by Kim et al. [32]. In a typical process, fruit water was removed from potatoes by grinding the tubers in a blender and decanting the liquid. Starch was extracted by centrifugation from the potato mash left after the removal of the fruit water. Crude starch was washed with water by making a slurry with approximately equal volumes of starch and water, followed by centrifugation and drying of the solid deposit, hereafter denoted as native potato starch.

#### 2.2.1. Supercritical CO_2_ Process

The supercritical CO_2_ procedure was conducted in a one-liter stainless-steel high-pressure autoclave mounted with a diaphragm-type compressor (Superpressure, Newport Scientific, Inc., Jessup, MD, USA) operating at up to 6000 psi, and a thermostatic bath to achieve and maintain the CO_2_ supercritical conditions (P > 1073 psi and T > 31 °C) as previously reported by Nlandu et al. [33]. The pressure in the reactor was regulated by a Tescom valve placed between the compressor and the reactor. A heat exchanger with an oil bath was located after the pump and it was used to heat the CO_2_ to reach the desired temperature. Thermostated liquid jacket was used to control the reactor temperature. All lines were electrically heated and insulated to keep the fluid temperature constant along the different sections. Pressures were measured by manometers and temperatures by thermocouples. In a typical run, the reactor was charged with native potato starch (1 g) and hermetically closed. The CO_2_ was aspirated from a cylinder furnished with a dip tube, pressurized to 2500 psi, and conducted to the reactor vessel. The reactor was heated to the desired temperature. Additional CO_2_ was regularly added to obtain the pre-set pressure value. After 4–8 h, the pressure was released to atmospheric pressure, and the solid product was collected. The tests were conducted under different conditions as presented in Table 1.

#### 2.2.2. High-Energy Ball Milling

The high-energy ball milling (HEBM) of potato starch was performed using the planetary ball milling system Emax from Retsch Company (Newtown, PA, USA). Potato starch granules were placed in a 50 mL stainless-steel reactor previously filled with 30 g of stainless-steel spherical balls with 5 mm diameters, either under dry conditions or in the presence of water (20 mL of distilled water). The high-energy ball milling treatment was performed at a rotation speed of 1100 r/min for 30 min at 75 °C as reported by Liu et al. [14] and Vertuccio et al. [20]. The resulting treated starch under dry conditions was directly sieved, whereas the starch samples treated under wet conditions were either dried in a vacuum oven at 40 °C for 12 h or dried via lyophilization, then ground and sieved.

#### 2.2.3. Ultrasonication

The high-intensity ultrasonic processor (450 W Model, 20 kHz, maximum wave amplitude of 210 µm and maximum nominal power of 450 W) from Branson Ultrasonics Corporation (Danbury, CT, USA) was used for the ultrasonication of the starch. In a typical test, starch (200 mg) and distilled water (4 mL) were mixed in a glass vial, then the ultrasonic radial probe (Sonotrode S3, 3 mm in diameter) was immersed in the starch suspension as reported in previous works [29,30]. Tests were also performed under dry conditions without adding water to the starch. The energy input was controlled by setting the amplitude of the sonication probe at 10% for 1 h. The resulting treated starch under dry conditions was directly sieved, whereas the starch samples treated in aqueous suspensions were either dried in a vacuum oven at 40 °C for 12 h or dried via lyophilization, then ground and sieved.

#### 2.2.4. Characterization of the Native and Treated Starch Samples

The powder XRD patterns of native and treated potato starch samples were obtained using a Rigaku D-Max-Ultima III (Rigaku Americas Corporation, The Woodlands, TX, USA) diffractometer with nickel-filtered Cu Kα radiation of wavelength 1.5406 Å. The X-ray generator was operated at 40 kV and 44 mA. The scanning regions of the diffraction angle 2*θ* were 5–55°, with a scan speed of 2 degrees/minute, covering all the significant diffraction peaks of starch crystallites. Duplicate measurements were made at ambient temperatures.

The degree of crystallinity of starch samples was quantitatively estimated as the ratio of the area of crystalline reflections to the overall diffraction area as previously reported [34,35,36]. A smooth curve that connected peak baselines was computer-plotted on the diffractograms. The area above the smooth curve was taken to correspond to the crystalline portion, and the lower area between the smooth curve and the linear baseline that connected the two points of intensity at 2*θ* of 55° and 5° was taken as the amorphous section. The upper diffraction peak area and total diffraction area over the diffraction angle of 5–55° 2*θ* were integrated on Smadchrom software (Morgan and Kennedy Research, Australia). The relative crystallinity was quantitatively estimated as a ratio of the crystalline area to the total area between 5–55° (2*θ*) [37]. The percentage crystallinity was calculated using the following equation [38]:

Crystallinity (%) = *I_c_*/(*I_a_* + *I_c_*) × 100

where *I_a_* is the amorphous area and *I_c_* is the crystalline area on the X-ray diffractogram.

The surface morphology of the native and different pretreated starches was examined using a scanning electron microscope (JEOL 840-A, Tokyo, Japan) operated at an accelerating voltage of 10 to 20 kV. Before analysis, the samples were prepared via sputter coating with platinum to obtain conductive surfaces. The microstructure and sizing of the potato starch samples recovered from the hydrolysates were investigated using a transmission electron microscope (JEOL JEM-1230, Tokyo, Japan) at an accelerating voltage of 80 kV. Before analysis, each representative sample was suspended in ethanol and sonicated for 5 min. Then, a drop of the suspension was placed on a carbon microgrid and dried at a temperature of 60 °C for 20 min.

#### 2.2.5. Enzymatic Hydrolysis

Enzymatic hydrolysis was performed on different starch samples. The effects of Pullulanase concentration (40, 80, 160, and 320 npun/g starch), temperature (20, 40, and 60 °C), and pH (4, 7, and 10) on the extent of starch hydrolysis were investigated. In a typical run, potato starch (100 mg) was mixed with 5 mL H_2_O, then, 500 μL of the starch solution (10 mg starch) was mixed with a predetermined volume of Pullulanase solution corresponding to 40–320 npun/g starch. The mixture was agitated at 130 rpm for a total duration of 6 h. New Pullulanase Unit Novo (npun) was defined as the amount of enzyme, that, under standard conditions, hydrolyzes pullulan, liberating reducing carbohydrates with reducing power equivalent to 1 µmole glucose per minute [39]. Aliquots of the different reaction media were withdrawn every 70 min and analyzed using size-exclusion chromatography with pulsed amperometric detection (SEC-PAD) and Pullulan standards as previously described [40]. Recovery of the resulting hydrolyzed starch materials in a powder form was performed through nanoprecipitation as reported by Nlandu et al. [41]. Hence, the enzymatic hydrolysis reaction was stopped and the suspension was put into a boiling water bath for 15 min to deactivate the enzyme. Ethanol, in a 1 to 10 ratio, was added to the gelatinized supernatant solution for 24 h at a temperature of 4 °C. Subsequently, the precipitation could be achieved via supersaturation, followed by nuclei and particle growth, leading to the formation of colloidal starch particles. The suspension was then centrifuged at 10,000 rpm at a temperature of 5 °C for 5 min and the obtained precipitate was freeze-dried. The obtained material was put in water (5%), reheated to a temperature of 120 °C for 30 min, stored at a temperature of 4 °C for 24 h, then oven-dried at a temperature of 30 °C for 24 h. Finally, the obtained material was sieved using a 140-mesh sieve to obtain the potato starch nanoparticles.

Size-exclusion chromatography (SEC) was performed using a Dionex chromatographic system ICS 2500 (ThermoFisher Scientific, Mississauga, ON, Canada) consisting of a gradient pump GP50, an electrochemical detector ED50, and a thin-layer type amperometric cell outfitted with a gold electrode and Ag/AgCl reference electrode. Pulsed amperometric detection (PAD) utilized a repeating sequence of three potentials, which were applied for specific durations. PAD is most sensitive to carbohydrates at a pH of 12 or greater, but a mobile phase of this pH would quickly destroy the polymer-based size-exclusion columns. Instead, a buffered mobile phase was used and sodium hydroxide was added post column to raise the pH. Two TSKgel size-exclusion columns G6000PWxl and G4000PWxl were used in tandem at 40 °C. The eluent was 10 mM acetate buffer (0.28 mL concentrated glacial acetic and 0.68 g sodium acetate trihydrate dissolved in 1.0 L of 18-MΩ water) at a flow rate of 0.35 mL/min and stored in a pressurized bottle with argon. The post column reagent was 300 mM sodium hydroxide (15 mL sodium hydroxide solution (50%) diluted to 1 L with 18-MΩ water) at 0.65 mL/min flow rate and stored in a pressurized bottle with argon. The sample volume injected was 25 μL. The Chromeleon software (ThermoFisher Scientific, Mississauga, ON, Canada) performed the calibration curve and integrated all the chromatographic data. The PeakFit software was used in the deconvolution procedure to automatically locate hidden peaks through a Gaussian response function with a Fourier deconvolution/filtering algorithm.

## 3. Results and Discussion

**Figure 1:**
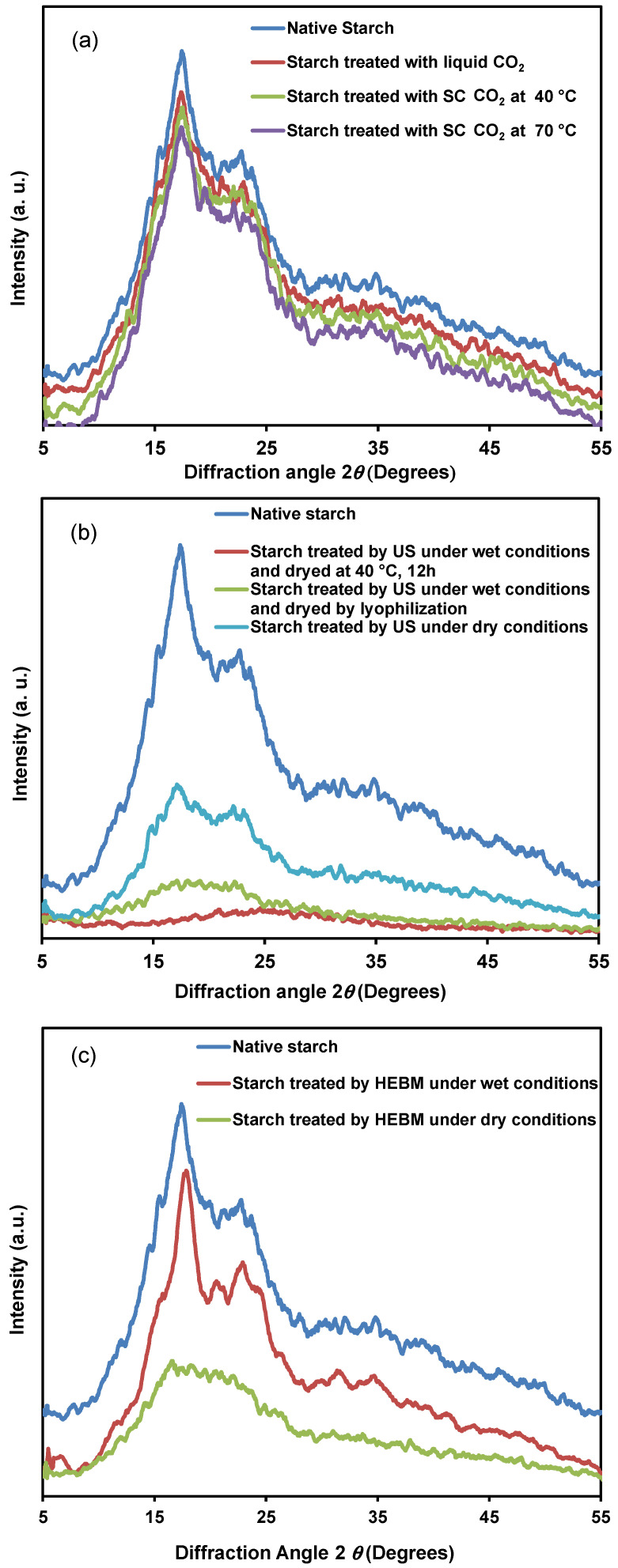
X-ray powder diffraction patterns of (**a**) native and treated starch using liquid and supercritical CO_2_; (**b**) native and treated starch using ultrasonication; (**c**) native and treated starch using HEBM.

The starch granules are semi-crystalline white particles, insoluble in water at room temperature. The size and shape of the granules are specific to each plant species. In a very simplified way, the organization of the starch grain results from the arrangement of amylose and amylopectin in amorphous and crystalline zones arranged concentrically from the hilum. The crystallinity of the starches is mainly due to the double helix chains of amylopectin and the cohesion of the crystalline zones is ensured by intermolecular hydrogen bonds [42]. The X-ray diffraction patterns of native and treated starch samples are presented in Figure 1. The corresponding X-ray diffraction parameters and crystallinity level calculated from the ratio of the diffraction peak area and the total diffraction area are given in Table 2. The scattering angle, at which the diffraction intensities can be observed, was 2*θ*, and the *d* spacing was used to discriminate the planes of different sites. Native and CO_2_-treated potato starches, as well as starch treated with HEBM, gave rise to the strongest diffraction peaks at around 17° 2*θ* (d = 5.058 Å) and a few small peaks at around 2*θ* values of 20° (d = 4.448 Å), 22° (d = 4.037 Å), and 23° (d = 3.855 Å). The X-ray powder diffraction patterns of Figure 1 are characteristic of potato starches associated with the B-type crystalline polymorph pattern. The relative crystallinity of the starches demonstrated a crystallinity ranging from 19 to 22% (Table 2), which agreed with previous data on potatoes [43]. No significant change in the type of crystalline pattern was observed as a result of CO_2_ treatment compared to the native starch, as the major peaks were similar. However, the relative intensity of the predominant diffraction peaks of starch granules decreased slightly after supercritical CO_2_ treatment at 70 °C corresponding to a decrease in crystallinity (Table 2). There was a decrease in the relative crystallinity percentage of starch granules treated with HEBM under dry conditions (Table 2). This may be due to the destruction of the crystalline portion within the cracked granules as evidenced in the SEM images (to be discussed below). On the other hand, starch granules mixed with water and treated with HEBM led to a higher percentage of relative crystallinity. The effect of ultrasound on the X-ray pattern and the degree of crystallinity of the potato starch granules are shown in Figure 1 and Table 2. After sonication, the diffraction peaks at 17°, 20°, 22°, and 23° strongly decreased in intensity for the sample treated under dry conditions. The effects were most noticeable for the starch mixed with water and dried under vacuum, with complete loss of diffraction peaks indicating that the crystal structure was destroyed after sonication and vacuum drying. The same pattern was observed for the starch mixed with water and lyophilized. It follows that the ultrasonic treatment had greatly disturbed the crystalline structure of the starch and led to the formation of either a transparent gel or a foam depending on the drying process as revealed by SEM and discussed later. 

**Figure 2:**
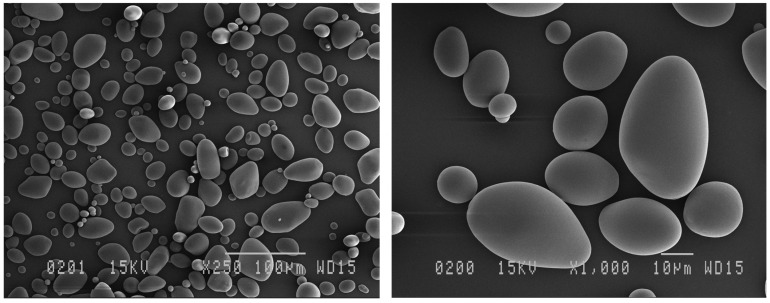
SEM images of native potato starch granules from the “Gabrielle” variety.

**Figure 3:**
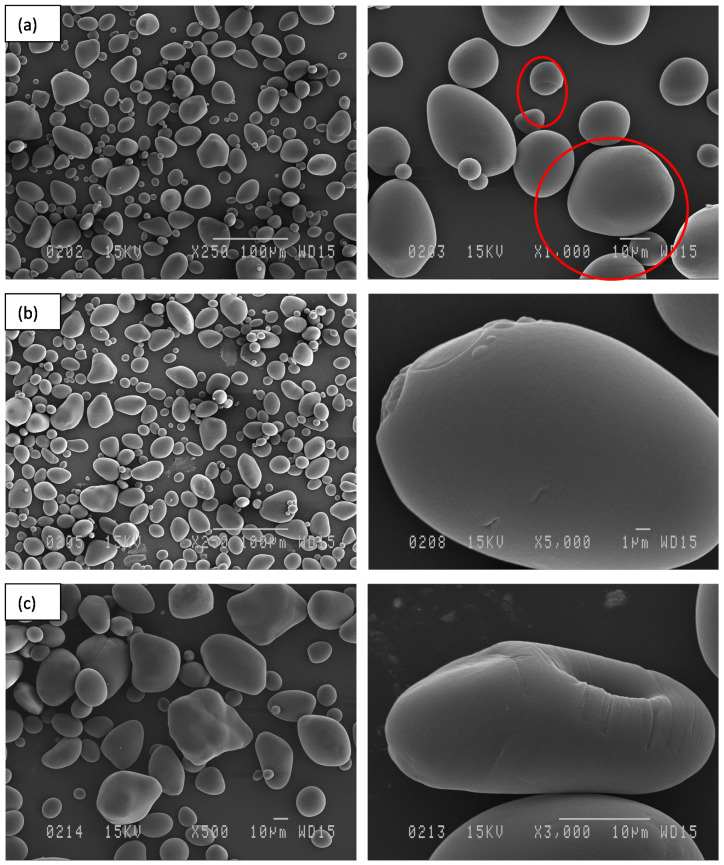
SEM images of potato starch treated with (**a**) liquid CO_2_ (irregular shapes of granules are circled in red); (**b**) supercritical CO_2_ at 40 °C; (**c**) supercritical CO_2_ at 70 °C.

The scanning electron micrographs of native potato starch of the Gabrielle variety are presented in Figure 2. Potato starch displayed spherical- or polygonal-shaped granules of the B-type with diameters less than 10 μm, and granules with diameters of about 5 μm were predominant. The morphology of the native starch granules shown in Figure 2 agreed with previous reports [44]. In addition, the treated starch samples exhibited various morphologies depending on the treatment. The starch treated with liquid CO_2_ exhibited irregularly shaped granules; however, the B-type form of the granules was maintained (Figure 3a). Morphological changes were visible in the starch sample treated with supercritical CO_2_ at 40 °C, consisting of small irregularly shaped granules with defects on the surface (Figure 3b). The starch sample treated with supercritical CO_2_ at 70 °C evidenced the formation of some cracks and pores on the granule surface (Figure 3c). Some of the deformed granules seemed to adhere to each other. The surface of the granules was rougher, and at higher magnification (×3000) the damage was clearly visible.

**Figure 4:**
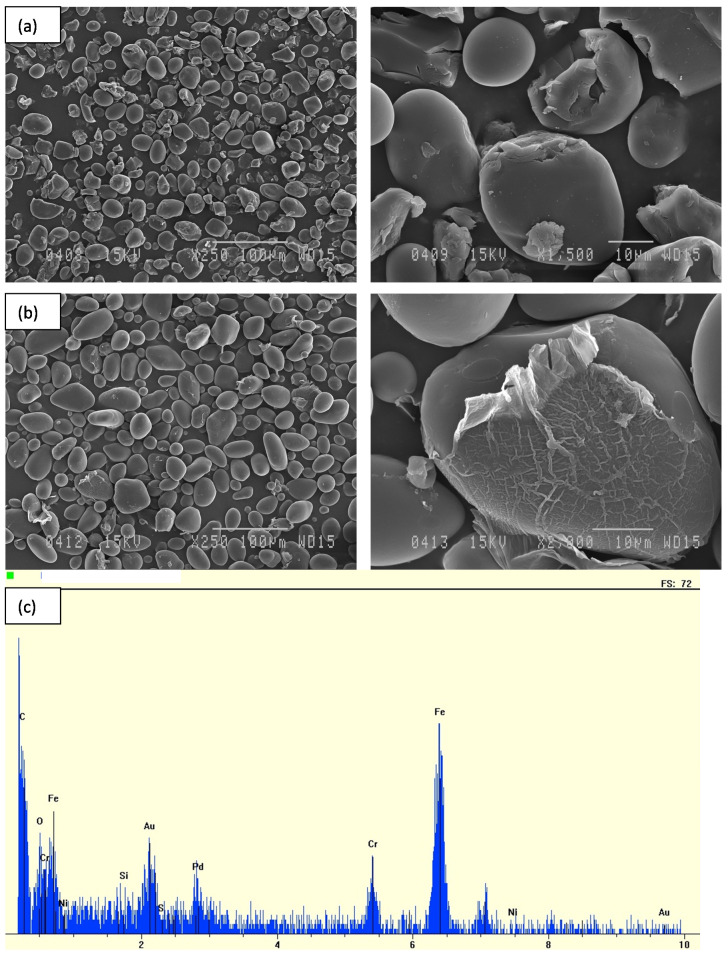
SEM images of potato starch granules treated using HEBM under dry (**a**) and wet (**b**) conditions; energy-dispersive X-ray spectroscopy of potato starch treated using HEBM (**c**).

HEBM treatment led to the breakage of the granules as depicted in Figure 4a. Alteration of the granules’ morphology decreased considerably by adding water (Figure 4b). In addition, the main drawback of HEBM is related to the presence of metal particles from the deterioration of the stainless-steel balls. This observation was confirmed by the analysis of metal particles by scanning electron microscopy with energy-dispersive X-ray spectroscopy. The result is shown in Figure 4c, with the chemical composition of the metal particles evidencing the occurrence of chromium, iron, and nickel stemming from the stainless steel.

**Figure 5:**
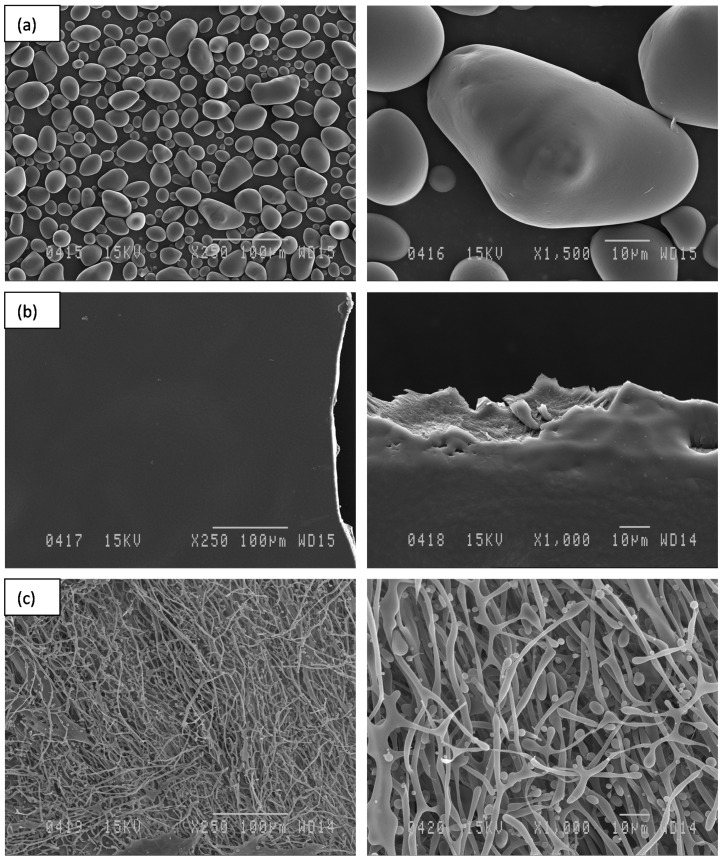
SEM images of potato starch granules treated with ultrasonication under dry conditions (**a**); under wet conditions and dried in a vacuum oven at 40 °C (**b**); under wet conditions and lyophilized (**c**).

**Figure 6:**
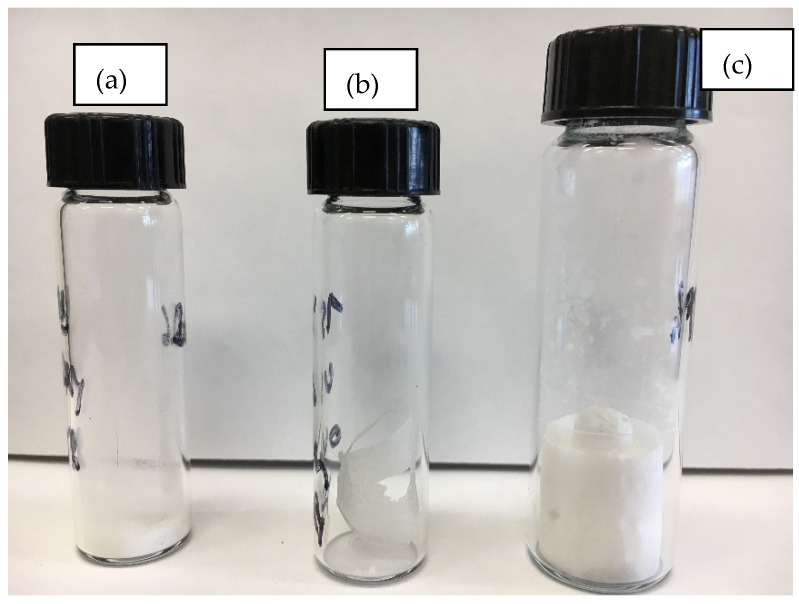
Different macro-morphologies of the potato starch from “Gabrielle” cultivar after treatment using ultrasonication under dry conditions (**a**); ultrasonication under wet conditions followed by drying in a vacuum oven at 40 °C (**b**); ultrasonication under wet conditions followed by drying by lyophilization (**c**).

The potato starch granules treated with ultrasonication under dry as well as wet conditions followed by different drying techniques exhibited various morphologies (Figure 5 and Figure 6). Under dry conditions, the effects of ultrasonication were weak and led to the deformation of the granules (Figure 5a), and the starch remained in a powder form (Figure 6a). In the presence of water and after drying in the vacuum oven at 40 °C, the starch granules formed a transparent gel (Figure 5b and Figure 6b). In the presence of water and after lyophilization, an unprecedented morphology was observed. Indeed, a foamy structure was obtained (Figure 6c), and the SEM image showed an expansion of the granules interconnected by filaments (Figure 5c). This is the first time that this type of starch morphology has been reported in the literature. Such a structure can be of great interest to the synthesis of biodegradable/ecofriendly cushioned packaging materials, medical tissue engineering applications, and the encapsulation/release of substances [45,46,47,48].

### Enzymatic Hydrolysis

**Figure 7:**
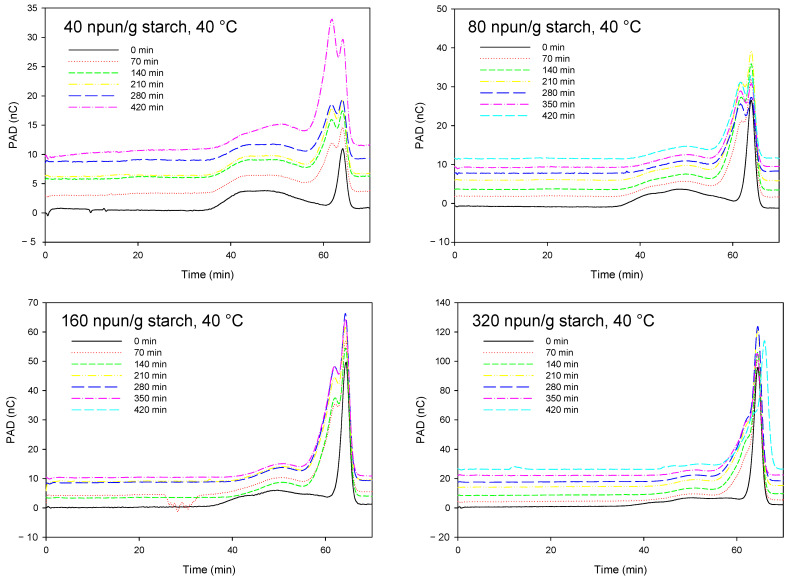
SEC-PAD chromatograms of the starch enzymatic hydrolysis kinetics with different concentrations of Pullulanase.

**Figure 8:**
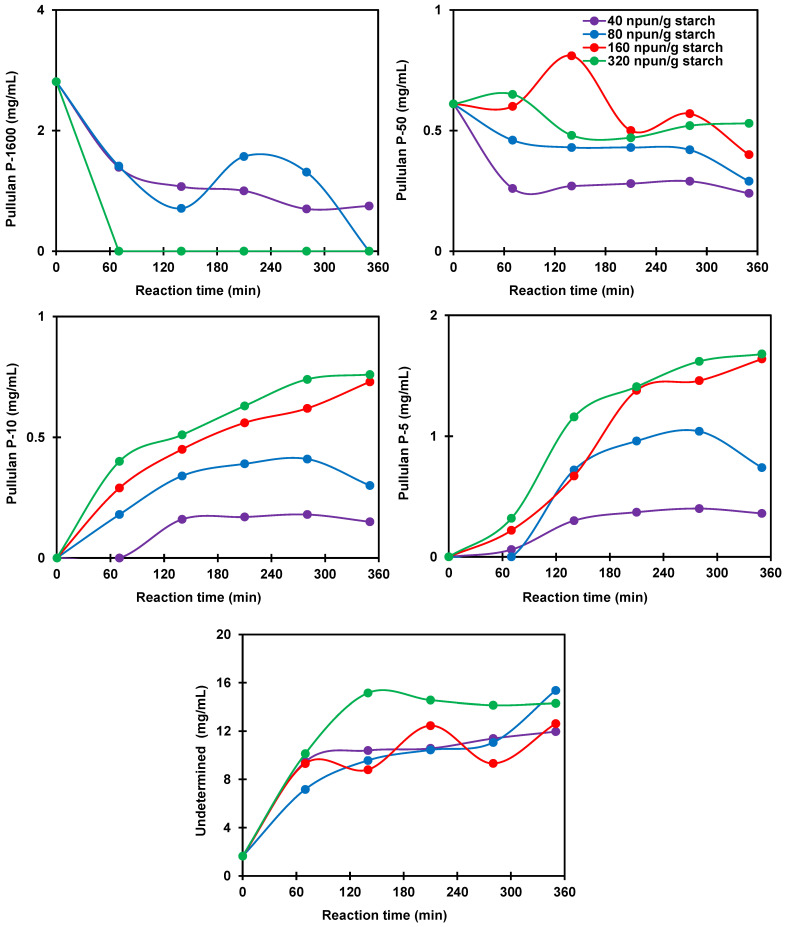
Effects of Pullulanase concentration on the production of hydrolysates containing molecules associated with Pullulan standard molecular weights P1600, P-50, P-10, P-5 and undetermined but lower than P-5 at 40 °C. Lines show trends.

The chromatograms in Figure 7 describe the kinetic profiles of the enzymatic hydrolysis as a function of the Pullulanase concentration (40, 80, 160, and 320 npun/g starch). The deconvolution of the peaks of these chromatograms allowed for the identification of the molecular weights of the resulting molecules following starch hydrolysis according to Pullulan standards. The time profiles of the different obtained fractions associated with the different Pullulan standards as a function of the concentration of the enzyme and for 6 h of reaction at 40 °C, in water, and agitation of 130 rpm are depicted in Figure 8. As seen, the hydrolysis of the potato starch was characterized by the formation of molecules with low molecular weights (undetermined but lower than P-5, P-5, and P-10 Pullulan standards), which increased with the concentration of Pullulanase. The concentration of the molecules with an intermediate molecular weight (P-50 Pullulan standard) was approximately stable during the reaction whatever the concentration of Pullulanase used. Indeed, these intermediate molecular weights were derived from the hydrolysis of large molecules and they were also hydrolyzed a second time, which leads to a roughly stable concentration profile. The hydrolysis of the molecules with a high molecular weight and associated with the Pullulan P-1600 standard was significantly depleted after only 70 min of reaction with Pullulanase concentrations of 160 and 320 npun/g starch, whereas it took 6 h with a concentration of 80 npun/g starch to reach similar results.

**Figure 9:**
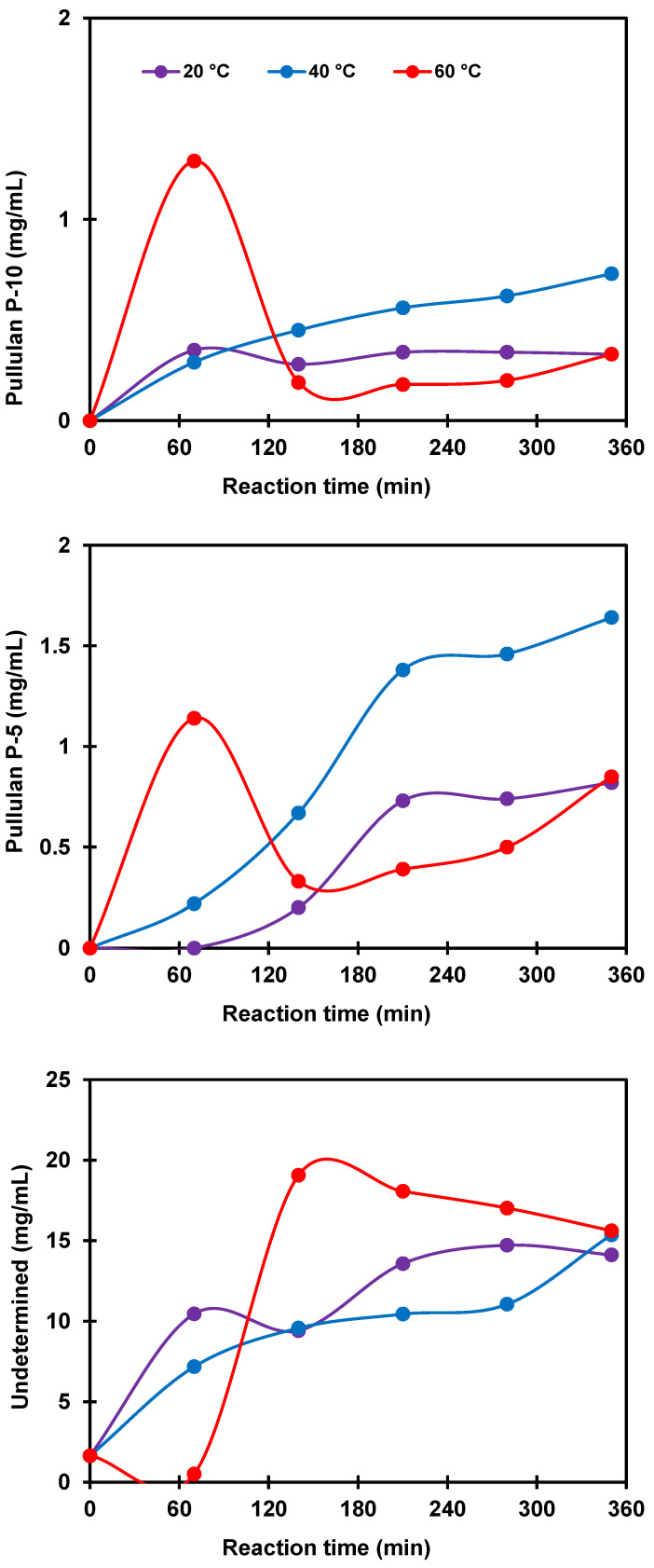
Effects of temperature on the production of hydrolysates containing molecules associated with Pullulan standard molecular weights P-10, P-5, and undetermined but lower than P-5 at 40 °C, 160 npun/g starch. Lines show trends.

Figure 9 depicts the effects of temperature on the enzymatic hydrolysis of potato starch. As revealed, an increase in the temperature from 20 to 60 °C led to the production of hydrolysates with increased concentrations of unidentified molecules with low molecular weight (<P-5 Pullulan standard). At a temperature of 60 °C and after 1 h of reaction, a peak concentration is observed for the molecules associated with the molecular weight of the P-5 standard, followed by a drastic decrease. The same phenomenon is noted with the concentration of the molecules associated with the P-10 standard. These observations suggest a high reactivity of the Pullulanase enzyme at 60 °C, resulting in the rapid production of molecules with a low molecular weight that were subsequently hydrolyzed.

**Figure 10:**
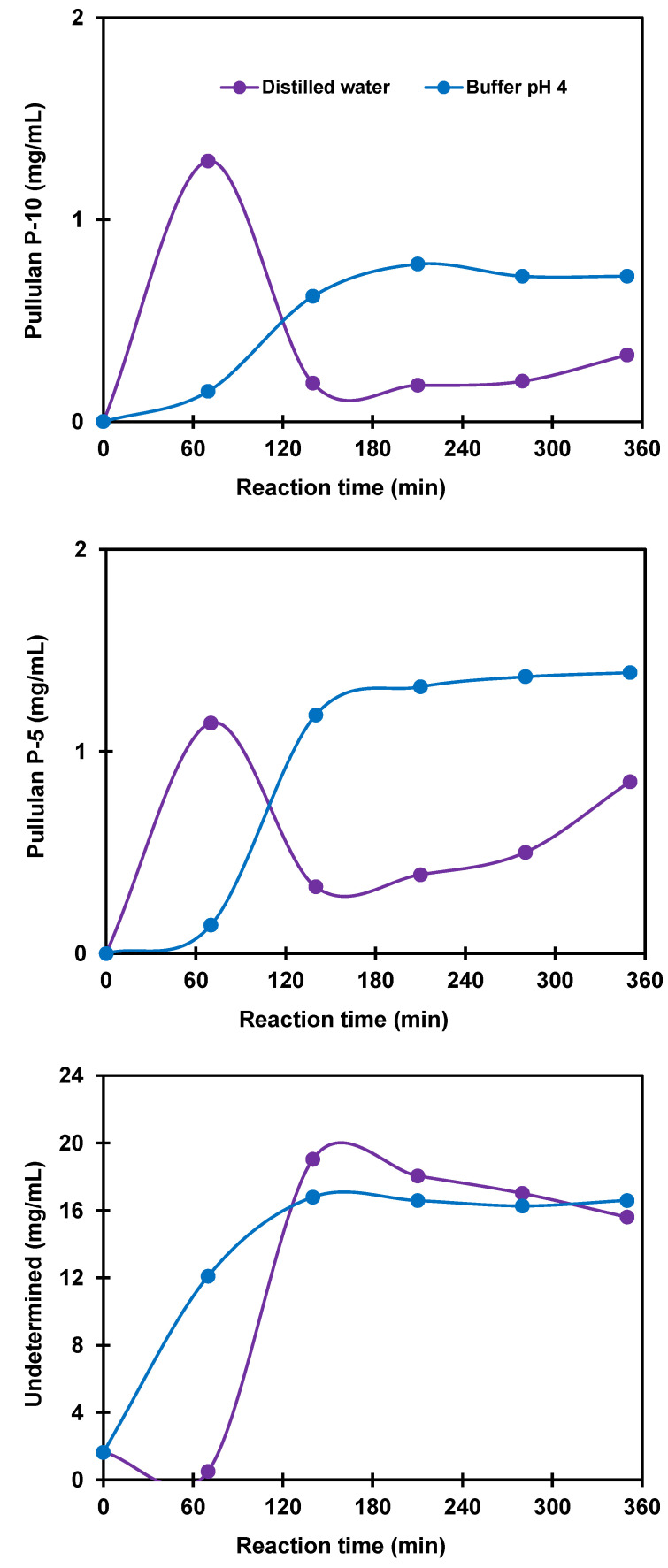
Effect of pH on the production of hydrolysates containing molecules associated with Pullulan standard molecular weights P-10, P-5, and undetermined but lower than P-5 at 40 °C, 160 npun/g starch. Lines show trends.

The pH of the solution is an important parameter in the investigated enzymatic hydrolysis of potato starch. Indeed, pH greatly influences the activity of the enzyme. In the present investigation, the effects of pH on the activity of Pullulanase for potato starch hydrolysis were studied using phosphate buffer solutions at pH 4 and 10 as well as distilled water. No activity of the Pullulanase enzyme was noticed at pH 10 in agreement with previously reported findings stating that Pullulanase performs best at pH 4–6 [49]. As displayed in Figure 10, the time profiles of the concentrations of the molecules with molecular weights corresponding to Pullulan standards P-10 and P-5, increased with reaction time when the hydrolysis was performed in a phosphate buffer at pH 4. On the contrary, those concentrations decreased when hydrolysis was performed in distilled water, thus confirming that the Pullulanase enzyme was more effective and its activity was more stable under acidic conditions.

**Figure 11:**
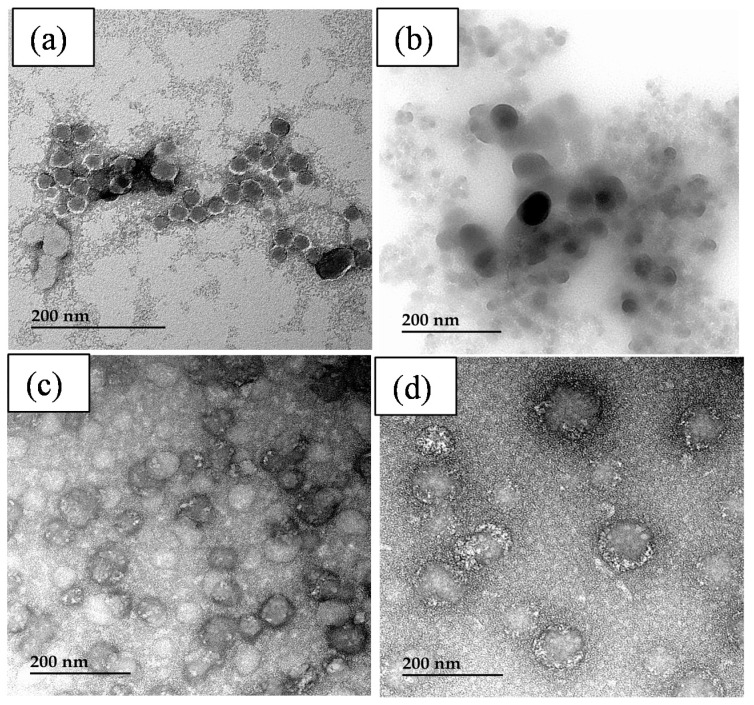
TEM images of starch particles stemming from hydrolysates of starch modified by supercritical CO_2_ (**a**); ultrasonication (**b**); HEBM under dry (**c**) and wet (**d**) conditions.

The TEM images of the potato starch particles recovered by the nanoprecipitation of the hydrolysates obtained after 6 h of reaction at 60 °C using Pullulanase at a concentration of 160 npun/g starch in a pH 4 buffer solution are illustrated in Figure 11. Before enzymatic hydrolysis, the different starch particles were previously modified either by supercritical CO_2_, ultrasonication, and HEBM under dry and wet conditions. As seen, spherical-like particles with very tiny diameters ranging from 20 to 50 nm were obtained.

## 4. Conclusions

The physical modification of potato starch granules was achieved using liquid and supercritical carbon dioxide, high-energy ball milling, and ultrasonication under dry and wet conditions. The differently treated potato starch granules were characterized morphologically using SEM and structurally using XRD. Following the supercritical and liquid CO_2_ treatments, XRD analysis revealed that the crystalline patterns of the treated potato starches were mainly unaffected. The effect of HEBM on the starch crystallinity was dependent on the conditions of milling. Dry conditions led to a decrease in crystallinity, whereas wet conditions resulted in the opposite effect. Ultrasonication caused an almost complete loss of starch crystallinity, which was confirmed by the drastic change in the starch morphology as revealed by SEM, which evidenced the formation of an unprecedented foamy morphology that could be useful for several innovative applications, such as the synthesis of biodegradable cushion packaging materials, medical tissue engineering, and drug delivery. As for the enzymatic hydrolysis of the starch using Pullulanase, it resulted in the production of hydrolysates containing starch polymers with low molecular weights corresponding mainly to P-10, P-5, and P < 5 Pullulan standards. The best results were obtained with a Pullulanase enzyme concentration of 160 npun/g of starch at a temperature of 60 °C in a pH 4 phosphate buffer solution. Among the different treatments investigated in the present work, only the enzymatic hydrolysis allowed the production of nanoparticles sized between 20 and 50 nm, which can be potentially useful for advanced applications, such as reinforcement in food packaging technology and carriers for the delivery of bioactive or therapeutic agents in the medical domain. This represents a judicious valorization of downgraded potatoes.

## Figures and Tables

**Table 1:** Operating conditions of supercritical CO_2_ procedure.

Starch Samples	Pressure (atm)	Temperature (°C)	Time (hours)
Liquid CO_2_	136	10	4
Supercritical CO_2_	136	40	8
Supercritical CO_2_	136	70	8

**Table 2:** X-ray powder diffraction of native and treated potato starch samples.

Potato Starch	Diffraction Peak at 2*θ* Value (° Angle)				Degree of Crystallinity (%)	Crystal Pattern
	17°	20°	22°	23°		
Native	33 *	23	23	21	19.4	B
	(5.058 Å) **	(4.448 Å)	(4.037 Å)	(3.855 Å)		
**Liquid and supercritical CO_2_**						
Liquid CO_2_ at 10 °C	32	25	18	25	19.5	B
	(5.089 Å)	(4.440 Å)	(4.039 Å)	(3.868 Å)		
Supercritical CO_2_ at 40 °C	34	19	22	25	19.7	B
	(5.062 Å)	(4.449 Å)	(4.038 Å)	(3.871 Å)		
Supercritical CO_2_ at 70 °C	27	25	25	23	19.0	
	(5.055 Å)	(4.447 Å)	(4.030Å)	(3.831Å)		
**High-energy ball milling**						
Dry conditions	26.4	24.9	25.4	23.3	14.3	
	(5.218 Å)	(4.430 Å)	(4.040 Å)	(3.867 Å)		
Wet conditions	28.6	22.2	23.5	25.7	22.2	B
	(5.250 Å)	(4.454 Å)	(4.039 Å)	(3.879 Å)		
**Ultrasonication**						
Dry conditions	28.8	23.2	24.2	23.8	13.8	B
	(5.214 Å)	(4.435 Å)	(4.027 Å)	(3.867 Å)		
Wet conditions after drying at 40 °C, 12 h	18.9	24.3	28.0	28.8	6.8	
	(5.219 Å)	(4.451 Å)	(4.045 Å)	(3.861 Å)		
Wet conditions after lyophilization	26.5	25.3	24.4	23.7	7.7	
	(5.228 Å)	(4.436 Å)	(4.026 Å)	(3.866 Å)		

* Relative intensity. ** Interplar spacing.

## Data Availability

All the data generated or analyzed within the present investigation are included in this manuscript.
